# Relation between *ADIPOQ* Gene Polymorphisms and Type 2 Diabetes

**DOI:** 10.3390/genes6030512

**Published:** 2015-07-08

**Authors:** Zhi-Peng Li, Mei Zhang, Jie Gao, Guo-Yan Zhou, Shuang-Qing Li, Zhen-Mei An

**Affiliations:** 1 Golden Card Hospital, West China Hospital, Sichuan University, Chengdu 610041, China; E-Mail: zhilipengirl@126.com; 2 Department of Laboratory Medicine, West China Hospital, Sichuan University, Chengdu 610041, China; E-Mail: zhangmaile@163.com; 3 Department of Endocrinology, West China Hospital, Sichuan University, Chengdu 610041, China; E-Mails: gaozhjiear@163.com (J.G.); zhougirlyan@126.com (G.-Y.Z.); anzhemaile@yeah.net (Z.-M.A.)

**Keywords:** *ADIPOQ*, type 2 diabetes, genetic polymorphism, case-control study

## Abstract

Objective: The manuscript investigates the relation between adiponectin gene (*ADIPOQ*) polymorphisms and type 2 diabetes mellitus (T2DM) in a Chinese population. Methods: We designed a case-control study involving 340 normal glucose tolerant (NGT) subjects and 340 type 2 diabetes patients. Three SNPs (rs182052, rs1501299, and rs7627128) were genotyped by TaqMan methods. Results: We found that rs7627128, rs1501299 and rs182052 were significantly associated with T2DM. Haplotypes analysis indicated that the frequency of the haplotypes A-A-T was frequent in T2DM patients (OR = 2.10; 95%CI: 1.44–2.90; *p* < 0.001), but G-A-T was more frequent in the control group than in the T2DM group (OR = 0.66; 95%CI: 0.54–0.81; *p* < 0.001). Conclusion: The *ADIPOQ* genetic polymorphisms were associated with type 2 diabetes in a Chinese population.

## 1. Introduction

The pathogenesis of type 2 diabetes mellitus (T2DM) remains unclear. However, previous studies suggested that T2DM is a complex disease resulting from the interaction between genetic polymorphisms and environmental factors [1]. Several genes have been identified as susceptible genes for T2DM, including the *C5L2* gene [2], *CYP2J2* gene [3] and *CCR5* gene [4,5]. However, these susceptible genes can only explain a small fraction of the susceptibility to T2DM.

Recently, adipose tissue was considered to play an important role in the pathogenesis of diabetes, as well as obesity by secreting a variety of secretory proteins [6]. Adiponectin is one of the major adipocyte secretory proteins most abundantly found in human plasma, with potent roles in insulin sensitivity in muscle and liver, regulating energy homeostasis and glucose tolerance [7,8]. Adiponectin is a product of the *ADIPOQ* gene, which is located on human chromosome 3q27, where a region composed of three exons that span 17 Kb, identified as a susceptibility locus for metabolic syndrome and T2DM, has been reported [9,10]. T2DM is a complex heterogeneous group of metabolic disorders, including hyperglycemia and impaired insulin action and/or insulin secretion, and a detailed etiology underlying T2DM is still unclear [11,12]. Therefore, it is necessary to identify the pathogenesis of T2DM. Recently, the *ADIPOQ* gene polymorphisms have been suggested to be implicated in the risk for type 2 diabetes; however, association studies have reported conflicting results [13,14]. Therefore, we designed a case-control study to derive an association between the *ADIPOQ* gene polymorphisms and T2DM risk in a Chinese population.

## 2. Subjects and Methods

### 2.1. Ethnics

The present study has been performed with the approval of the ethics committee of West China Hospital, Sichuan University, and is in compliance with the Helsinki Declaration. Informed consent for the study was collected from all of the candidate subjects.

### 2.2. Subjects

A total of 680 participants, including 340 T2DM patients and 340 healthy control subjects (normal glucose tolerant (NGT)), were selected for the present study from January 2010 to June 2014. Diabetes was confirmed according to the World Health Organization (WHO) consulting group criteria, for which an oral glucose tolerance test has a 2-h plasma glucose value ≥11.1 mmol/L (200 mg/dL); and the subjects with 2-h plasma glucose value <7.8 mmol/L (140 mg/dL) were labeled as NGT [15]. The characteristics of the case and control subjects are shown in Table 1.

**Table 1:** Characteristics of the participants.

Groups	N	Sex (Male/Female)	Age (years)	BMI (kg/m^2^)	Glucose (mmol/L)	TG (mmol/L)	TC (mmol/L)	HDL-C (mmol/L)	LDL-C (mmol/L)
T2DM group	340	240/100	54.2 ± 11.2	24.7 ± 2.4	5.44 ± 0.41	1.77 ± 0.21	2.90 ± 0.69	0.88 ± 0.21	1.44 ± 0.41
Control group	340	244/96	54.1 ± 10.4	23.1 ± 1.6	4.26 ± 0.43	1.43 ± 0.15	2.56 ± 0.54	0.94 ± 0.20	1.20 ± 0.31
*p*		0.901	0.432	<0.001	<0.001	<0.001	<0.001	0.771	<0.001

### 2.3. Phenotype Measurements

We collected clinical data, such as weight, height, waist circumference and other data. The BMI was calculated as weight (in kg) divided by the square of height (in m). Fasting plasma glucose, serum cholesterol, serum triglycerides, high-density lipoprotein cholesterol and low-density lipoprotein cholesterol were measured as described in a previous protocol [16]. Glycated hemoglobin (HbA1c) was estimated by high performance liquid chromatography using a Variant™ machine (Bio-Rad, Hercules, CA, USA). Serum insulin concentration was estimated using an enzyme-linked immunosorbent assay (Dako, Glostrup, Denmark). Total serum adiponectin was measured by radioimmunoassay.

### 2.4. Genetic Analysis

Although there are 683 SNPs for the human *ADIPOQ* gene listed in the National Center for Biotechnology Information SNP database (http://www.ncbi.nlm.nih.gov/SNP), we only selected three SNPs (rs182052, rs1501299, and rs7627128) in the present study according to the methods described previously [17]. These three SNPs are all tagSNPs of the *ADIPOQ* gene, which can represent the genetic information of the other SNPs in the *ADIPOQ* gene. Genomic DNA was extracted from the whole blood by the phenol-chloroform method of DNA extraction. Genotyping was confirmed by the TaqMan method as described previously [18].

### 2.5. Statistical Analysis

We used SPSS 17.0 for Windows (SPSS, Chicago, IL, USA) to perform the statistical analysis. Hardy-Weinberg equilibrium (HWE) was tested using a χ^2^ test. Comparison of the means between the two groups was analyzed by Student’s *t* test. The χ^2^ test was used to compare the proportions of genotypes or alleles. We used the SHEsis software (http://analysis2.bio-x.cn/myAnalysis.php) [19,20] to perform the linkage disequilibrium (LD) analysis and haplotype construction. In the haplotype-based case-control analysis, haplotypes with a frequency of <0.03 were excluded. Statistical significance was established at *p* < 0.05.

## 3. Results

The genotype distribution of each SNP did not show a significant difference from the Hardy-Weinberg equilibrium values. As shown in Table 2 for total participants, the genotype and the allele frequency of rs182052, rs1501299 and rs7627128 were significantly different between the T2DM patients and the control subjects. According to the |D'| and r^2^ values, we considered that these three SNPs (rs1501299, rs182052 and rs7627128) are located in one haplotype block. In the haplotype-based case-control analysis, haplotypes were established through the use of all three SNPs. As shown in Table 3, the haplotypes A-A-T was frequent in T2DM patients (OR = 2.10; 95%CI: 1.44–2.90; *p* < 0.001), but G-A-T was lower in the T2DM patient group than in the control group (OR = 0.66; 95%CI: 0.54–0.81; *p* < 0.001).

**Table 2:** Distributions of the ADIPOQ genotype.

Groups	N	SNP	Genotypes (n, %)			*p*-Value	Allele		OR (95% CI)	*p*-Value
		rs182052	AA	AG	GG					
T2DM group	340		66 (19.41)	172 (50.59)	102 (30.0)	0.034	304 (0.450)	376 (0.550)	1.28 (1.05–1.59)	0.022
Control group	340		57 (16.76)	151 (44.41)	132 (38.82)		265 (0.390)	415 (0.610)		
		rs1501299	AA	AG	GG					
T2DM group	340		57 (16.76)	202 (59.41)	81 (23.82)	0.021	316 (0.466)	314 (0.534)	1.31 (1.04–1.65)	0.002
Control group	340		55 (16.18)	172 (50.59)	113 (33.24)		282 (0.410)	398 (0.590)		
		rs7627128	AA	AC	CC					
T2DM group	340		42 (12.35)	197 (57.94)	101 (29.71)	<0.001	281 (0.410)	399 (0.590)	1.43 (1.17–1.98)	0.002
Control group	340		37 (10.88)	153 (45.00)	150 (44.12)		227 (0.330)	453 (0.670)		

**Table 3:** Haplotype analysis results.

Variables	Case (n, Frequency)	Control (n, Frequency)	*p*-Value	OR (95% CI)
A-A-C	252 (0.37)	242 (0.36)	0.531	1.05 (0.84–1.27)
A-A-T	64 (0.09)	33 (0.05)	<0.001	2.10 (1.44–2.90)
G-A-T	151 (0.22)	205 (0.30)	<0.001	0.66 (0.54–0.81)
G-G-C	15 (0.02)	16 (0.02)	0.966	0.98 (0.55–1.75)
G-G-T	191 (0.28)	177 (0.26)	0.221	1.12 (0.91–1.35)

## 4. Discussion

In the present study, we found that the *ADIPOQ* gene rs1501299, rs182052 and rs7627128 polymorphisms were significantly associated with T2DM in a Chinese population.

T2DM is a complex disorder that may result in the interaction between genetics and environmental factors. There were many genes, such as Calpain 10, eNOS, the CRP gene, *etc.* [21,22,23,24,25], that have been found to be associated with T2DM. Previously, Ramya *et al.* found that the adiponectin gene variants and haplotype contribute to the genetic risk towards the development of type 2 diabetes, obesity and hypoadiponectinemia in a south Indian population [26]. Chung *et al.*, reported that *ADIPOQ* genetic polymorphisms rs2241766 (+45T > G), rs1063537, rs2241767 and rs2082940 were correlated with the progression of diabetic nephropathy (DN) in Taiwanese male patients with T2D [27]. Du *et al.* observed that *ADIPOQ* gene polymorphisms (rs266729, rs1063539, rs16861205 and rs7649121) were associated with increased risk for the T2DM in a Chinese population [28]. Our study also indicated that *ADIPOQ* gene polymorphisms were associated with T2DM in a Chinese population.

In addition, we carried out a haplotype-based case-control study to investigate the association of *ADIPOQ* polymorphisms with T2DM, and we found that rs182052, rs1501299 and rs7627128 polymorphisms were significantly associated with T2DM. Haplotype analysis suggested that the haplotype A-A-T and G-A-T was associated the increased risk or decreased risk for T2DM, respectively.

## 5. Limitations

Several limitations in the present study should be mentioned. Firstly, we did not compare the difference in serum adiponectin levels among different genotypes. Secondly, we did not detect the HOMA-IR [HOMA-IR is an index used to evaluate an individual’s level of insulin resistance. Calculated as follows: Fasting plasma glucose levels (FPG, mmol/L) × Fasting insulin (FINS, mIU/L)/22.5] in our study. Finally, the relatively small sample size may overestimate the OR value during the analysis of the risk for T2DM.

## 6. Conclusions

In conclusion, the present results indicate that T2DM is associated with the *ADIPOQ* gene polymorphisms. The A-A-T haplotype appear to be a useful genetic marker, and the G-A-T haplotype might be a protective factor from T2DM in Chinese people.
